# Classical galactosemia: neuropsychological and psychosocial functioning beyond intellectual abilities

**DOI:** 10.1186/s13023-019-1277-0

**Published:** 2020-02-07

**Authors:** Mendy M. Welsink-Karssies, Kim J. Oostrom, Merel E. Hermans, Carla E. M. Hollak, Mirian C. H. Janssen, Janneke G. Langendonk, Esmee Oussoren, M. Estela Rubio Gozalbo, Maaike de Vries, Gert J. Geurtsen, Annet M. Bosch

**Affiliations:** 1 Department of Pediatrics, room H7-270, Amsterdam University Medical Centre, MC, PO BOX 22660, 1100 DD Amsterdam, The Netherlands; 2 Psychosocial Department, Emma Children’s Hospital, Amsterdam UMC, University of Amsterdam, Amsterdam, The Netherlands; 3 Department of Medical Psychology, Amsterdam UMC, University of Amsterdam, Amsterdam, The Netherlands; 4 Department of Internal Medicine, Division of Endocrinology and Metabolism, Amsterdam UMC, University of Amsterdam, Amsterdam, The Netherlands; 5 Department of Internal Medicine, Radboud University Medical Center, Nijmegen, The Netherlands; 6 Department of Internal Medicine, Center for Lysosomal and Metabolic Diseases, Erasmus MC, University Medical Center Rotterdam, Rotterdam, The Netherlands; 7 Department of Pediatrics, Center for Lysosomal and Metabolic Diseases, Erasmus MC, University Medical Center Rotterdam, Rotterdam, The Netherlands; 8 Department of Pediatrics and Department of Clinical Genetics, Maastricht University Medical Center, Maastricht, The Netherlands; 9 Department of Pediatrics, Radboud University Medical Center, Nijmegen, The Netherlands

**Keywords:** GALT deficiency, Neuropsychology, Cognitive functioning, Intelligence, Behavior, Social functioning

## Abstract

**Background:**

Despite early diagnosis and treatment, Classical Galactosemia (CG) patients frequently develop long-term complications, such as cognitive impairment. Available literature primarily reports on general intellectual abilities and shows a substantially lower Full Scale Intelligence Quotient (FSIQ) in CG patients than in the general population. Both problems in social functioning as well as internalizing problems are often reported in CG patients. The combination of intelligence, cognitive functioning, behavior and social functioning has not been studied systematically in CG patients.

**Methods:**

To determine if CG patients demonstrate a specific neuropsychological and psychosocial profile, we investigated intelligence, functioning on multiple cognitive domains, behavior and social functioning with a comprehensive neuropsychological test battery and questionnaires (self- and proxy-reported).

**Results:**

The data of 48 patients, aged 4–47 years are reported. FSIQ ranged from 45 to 103 (mean 77 ± 14). A negative correlation between age and FSIQ was demonstrated (*p* = 0.037) which resulted directly from the inclusion of four young ‘milder’ patients detected by newborn screening (NBS) with an expected better clinical outcome. Compared to normative data, patients had significantly lower but highly variable scores on all cognitive domains, especially on tests requiring mental speed. In the context of the FSIQ, 43% of the cognitive test results exceeded IQ based expectations. Overall, the patients’ scores on social functioning were in the normal range but internalizing problems were frequently reported. In our cohort, an early initiation of dietary treatment due to NBS or family screening did not result in a more favorable neuropsychological outcome.

**Conclusions:**

In this study, we demonstrated that as a cohort, CG patients have a below average intelligence and impaired cognitive functioning without a distinctive neuropsychological profile. The effect of age on neurocognitive functioning should be assessed in longitudinal studies. Social functioning was not impaired, but patients may be at risk for internalizing problems. Considering the large variability in cognitive, behavioral and social functioning and the finding that cognitive outcomes may exceed IQ based expectations, an individual evaluation and follow-up is warranted in all CG patients to ensure timely support if needed.

## Background

Classical Galactosemia (CG, OMIM 230400) is an autosomal recessive inborn error of galactose metabolism, caused by a deficiency of the enzyme galactose-1-phosphate uridylyltransferase (GALT, EC 2.7.7.12). The ingestion of galactose from breastmilk or infant formula in the first weeks of life causes critical illness in affected neonates. A lifelong galactose restricted diet is the only available treatment which is lifesaving in the newborn period but does not prevent long-term complications such as cognitive impairment, speech- and language deficits and movement disorders [1–3]. A published systematic review and meta-analysis demonstrated a substantially lower Full Scale Intelligence Quotient (FSIQ) in CG patients compared to the general population with large individual differences and a FSIQ ranging from fully normal to severely impaired [4]. The cognitive outcome of CG patients reported in the literature is mainly based on intelligence tests that lead to IQ. However, IQ is principally a dimension of individual differences in overall cognitive functioning called ‘general intelligence’. It is the ultimate resultant of underlying more specific abilities. These abilities are referred to as cognitive functioning and encompass domains such as information processing speed, attention, memory, visuospatial functioning and executive functioning. Previous studies reporting on cognitive functioning in CG patients demonstrated below average to low functioning on several cognitive domains [5–9]. However, the outcomes on the cognitive domains differed between studies and results must be interpreted with care because studies mostly addressed only one cognitive domain, used one single test per cognitive domain and/or included small cohorts. A recently published systemic review demonstrated large differences between patients, but also suggested that specific cognitive impairments may cause the lower level of intellectual functioning observed in CG patients [10]. In order to investigate this properly, a comprehensive neuropsychological assessment addressing multiple cognitive domains with multiple tests per domain should be performed.

Besides the cognitive difficulties, problems in social functioning such as difficulties in making friends and maintaining a stable relationship, as well as internalizing behavior problems are often reported in CG patients and affect quality of life [2, 9, 11–13]. It has been suggested that CG patients exhibit autistic traits, however this has not been studied systematically and should be investigated in combination with social and behavioral functioning.

In order to provide patients with optimal support, more insight into the neurocognitive, social and behavioral functioning of CG patients is warranted. The aim of this study was to investigate the neuropsychological functioning of CG patients by assessing the combination of general intelligence, cognitive functioning on multiple domains, social functioning and behavior and in a well-documented cohort of pediatric, adolescent and adult patients with CG. The effect of an early initiation of dietary treatment on neuropsychological functioning will be evaluated as well.

## Results

Of 67 CG patients visiting our multidisciplinary galactosemia expertise outpatient clinic and 6 CG patients treated in other metabolic centers, 54 patients received a neuropsychological assessment. Six patients were excluded because patients did not consent with the use of their clinical data for research purposes (*n* = 3), had a second diagnosis influencing intellectual outcome (*n* = 1), the administered tests were not part of our standardized assessment (*n* = 1) and only a partly neuropsychological assessment was available due to visual impairment (*n* = 1).

### Demographics

In total, data of 48 patients are reported and demographics are presented in Table 1. The GALT erythrocyte activity was unknown in six patients with classical phenotypes. Our cohort includes four variant patients detected since the implementation of CG in the Dutch newborn screening (NBS) program in 2007 with residual erythrocyte GALT activity up 10% and possibly a better clinical outcome [14]. Two patients are homozygous for the p.Ser135Leu mutation with GALT deficiency in erythrocytes but residual GALT enzyme activity in other tissues which may improve clinical outcome [15]. The two homozygous p.Ser135Leu patients in our cohort were diagnosed late, at the age of 7 months and 10 years respectively. In the pre-NBS group (*n* = 30) (diagnosis based on clinical symptoms), with the exception of the late diagnosed p.Ser135Leu patients, the diet was started at a median age of 10 days [6, 39]. In the early treated group (*n* = 18) (diagnosis by NBS or family screening), the diet was started at a median age of 5.5 days (0-8).

**Table 1:** Patient Demographics

Variable		All CG patients	
Gender, *n* (%)		Female: 28 (58) Male: 20 (42)	
Age (years)		Median 16 (4–47)	
GALT erythrocyte activity (%), *n* (%)			
- < 3.3 - 3.3–8.7 - Unknown		36 (75) 6 (12.5) 6 (12.5)	
Diagnosis based on (*n*):			
- Clinical symptoms (pre-NBS) - NBS - FS		30 12 6	
General Intelligence (*n* = 48)			
FSIQ VIQ PIQ		Mean 77 (95% CI 72–80), SD 14 Mean 82 (95% CI 77–85), SD 15 Mean 78 (95% CI 73–81), SD 15	
FSIQ, *n* (%)			
- FSIQ < 70 - FSIQ ≥ 70–85 - FSIQ > 85–100 - FSIQ > 100		12 (25) (group 1) 20 (42) (group 2) 15 (31) (group 3) 1 (2) (group 3)	
Wechsler scales (*n*)	FSIQ	VIQ	PIQ
- WPPSI-IIINL (7) - WISC-IIINL (19) - WAIS-IVNL (22)	Mean 84, SD 11 Mean 79, SD 16 Mean 74, SD 13	Mean 84, SD 17 Mean 82, SD 16 Mean 81, SD 14	Mean 94, SD 6 Mean 76, SD 15 Mean 74, SD 13
Educational level, *n* (%)			
- Elementary school (*n* = 48)			
- Normal education - Special education - Unknown		25 (52) 15 (31) 8 (17)	
- Secondary school (*n* = 32)			
- Normal education - Special education - Unknown - Not applicable (age < 12y)		16 (50) 9 (28) 7 (22) 16 (33)	
- Educational attainment patients^a^	*n* = 17	Educational attainment Dutch population ^b^	
1. Low educational level 2. Secondary educational level 3. High educational level Unknown	35% 47% 6% 12%	1.Low educational level 2. Secondary educational level 3. High educational level Unknown	31.5% 38.5% 29% 1.5%

*CG* classical galactosemia, *GALT* galactose-1-phosphate uridyltransferase, *NBS* newborn screening, *FS* family screening, *FSIQ* full scale IQ, *VIQ* verbal IQ, *PIQ* performal IQ, *WPPSI* Wechsler Preschool and Primary Scale of Intelligence, *WISC* Wechsler Intelligence Scale for Children, *WAIS* Wechsler Adult Intelligence Scale. ^a^ Highest level of completed education, ^b^ Data from the Dutch National Bureau of Statistics

### Educational attainment

A total of 15 out of 48 patients (31%) attended or attend to date special schools for primary education compared to 4.5% in the general population [16] (Table 1). Of the patients aged 12 years and older who completed primary education, 9/32 (28%) attend or attended special schools for secondary education compared to 3.0% in the general population [16]. In the Netherlands, one of the eligibility criteria for special education (smaller classes and tailored education) is a FSIQ below 80.

Of the patients who completed their education, 6/15 completed education at a low educational level (of which five completed special education), 8/15 completed education at the secondary vocational level and 1/15 completed education at a high educational level, which is lower when compared to the general population (Table 1).

### General intelligence

The FSIQ ranged from 45 to 103, with a mean of 77 (Table 1). The FSIQ did not significantly differ between males and females, nor between children and adults. Age was significantly correlated with FSIQ (F (1, 46)=4.62, β-0.42 (95%CI -0.82 –  -0.03), *p* = .037).

The results of the VIQ, PIQ and FSIQ of the Wechsler Scales of Intelligence are listed in Table 1. In 11 out of 48 patients, there was a significant difference of 15 or more points between the PIQ and VIQ. In seven patients (five adults and two children), this was in favor of the VIQ and the gap between VIQ and PIQ ranged from 15 to 24 IQ points. In four patients (all children), this was in favor of the PIQ and the gap between PIQ and VIQ ranged from 15 to 30 IQ points.

#### FSIQ and educational attainment

In 15 adult patients, the highest level of completed education was reported and in 5/15 (33%) this was special education, while 13/15 (85%) had a FSIQ below 80. Of the two patients with a FSIQ above 80, one patient completed secondary vocational education (FSIQ 81, 95%CI 77–87) and one patient completed higher professional education (FSIQ 88, 95%CI 83–93).

#### FSIQ and the initiation of treatment

There was no significant difference in VIQ, PIQ and FSIQ between patients in the pre-NBS group (*n* = 30) and early treated patients (*n* = 18). The exclusion of the late diagnosed p.Ser135Leu patients (*n* = 2) and NBS detected variant patients (*n* = 4) did not change these results.

### Cognitive functioning

The cognitive functioning test results are reported in Table 2. The reported T-scores of patients were compared to normative T-scores, based on a normative population sample.

**Table 2:** Cognitive Functioning Results

Domain	*N*	Results patients	*P*-value^a^	*N*	FSIQ 50–69 (Group 1)	*N*	FSIQ 70–85 (Group 2)	*N*	FSIQ > 85 (Group 3)	*P*-value^b^
Learning & Memory										
- AVLT Immediate Recall - AVLT Delayed Recall - AVLT Delayed / Immediate - Digit span	19 19 19 35	46.0 (9–61) 46.0 (15–65) 52.0 (38–65) 43.0 (20–63)	**0.029** 0.545 0.445 **< 0.0005**^**c**^	4 4 4 8	40.0 (9–46) 49.5 (15–58) 55.5 (43–62) 30.00 (20–57)	11 11 11 15	45.00 (35–61) 46.00 (34–65) 52.00 (38–65) 43.00 (27–60)	4 4 4 12	49.50 (47–51) 50.00 (43–55) 52.00 (39–62) 48.50 (33–63)	0.121 0.956 0.803 **0.017**
Visuospatial functioning										
GIT-2 spatial test Block design	19 42	36.0 (23–50) 38.5 (20–53)	**< 0.0005**^**c**^ **< 0.0005**^**c**^	4 9	26.5 (23–40) 30.0 (20–40)	11 17	35.0 (28–40) 37.0 (27–50)	4 16	41.5 (40–50) 37.0 (33–53)	**0.019** **< 0.0005**^**c**^
Executive functioning										
*Inhibition*										
- Stroop III (Inhibition) - Stroop III/II (Interference)	25 25	45.0 (20–56) 49.0 (30–66)	**0.003**^**c**^ 0.537	6 6	27.0 (20–49) 43.0 (30–60)	13 13	48.00 (22–56) 50.00 (31–66)	6 6	46.0 (35–53) 47.5 (40–63)	0.078 0.642
*Cognitive flexibility*										
- WCST Total number of errors - WCST Perseverative responses - WCST Percent Cconceptual level responses	24 24 24	50.5 (27–67) 51.0 (30–81) 49.5 (27–64)	0.988 0.626 0.951	6 6 6	46.0 (27–50) 46.0 (33–52) 48.0 (27–51)	12 12 12	51.50 (37–67) 53.00 (30–81) 52.00 (39–64)	6 6 6	52.0 (39–64) 52.0 (35–73) 51.0 (37–62)	0.134 0.278 0.270
Responses										
- TMT B/A - Letter fluency	25 19	44.0 (27–57) 37.0 (27–67)	**0.002**^**c**^ **0.001**^**c**^	6 4	45.0 (27–50) 31.0 (28–38)	13 11	43.00 (27–57) 39.00 (31–67)	6 4	48.5 (40–57) 34.0 (27–56)	0.510 0.143
Mental Speed										
- Stroop I (Color naming) - Stroop II (Word reading) - TMT A (Digit sequencing) - TMT B (Digit-Letter-Switching) - Symbol search - Substitution	25 25 25 25 41 42	40.0 (25–61) 37.0 (20–56) 52.0 (20–67) 45.0 (20–58) 43.0 (20–67) 40.0 (23–57)	**0.001**^**c**^ **< 0.0005**^**c**^ **0.352** **0.003**^**c**^ **< 0.0005**^**c**^ **< 0.0005**^**c**^	6 6 6 6 9 9	35.0 (25–43) 30.0 (20–40) 50.5 (20–59) 33.5 (20–47) 23.0 (20–50) 30.0 (23–47)	13 13 13 13 17 17	43.00 (35–61) 43.00 (20–56) 56.00 (43–67) 46.00 (20–58) 40.00 (27–67) 40.00 (30–57)	6 6 6 6 15 16	46.5 (33–55) 39.0 (33–56) 47.5 (33–67) 45.0 (33–56) 47.0 (40–60) 43.0 (33–53)	0.077 0.063 0.173 0.111 **0.001**^**c**^ **0.006**

Data reported in T-scores, median (ranges). ^a^ Patient data vs. normative data (T-score 50), ^b^ Comparison between FSIQ groups, ^**c**^ Significant after Bonferroni-Holm correction. *FSIQ* full scale IQ, *AVLT* auditory verbal learning test, *GIT-II* groninger intelligentie test 2, *Stroop* stroop color word test, *WCST* wisconsin card sorting test, *TMT* trail making test

#### Learning & Memory

On the AVLT, patients demonstrated lower scores on Immediate Recall, however the difference was not statistically different after correction for multiple testing. On the other two subtests, Delayed Recall and Delayed/Immediate Recall scores were comparable. On the Digit Span subtest, which requires auditory verbal memory and verbal working memory, patients had significantly lower scores.

#### Visuospatial functioning

The visuospatial functioning of patients was assessed with the GIT-2 spatial test and Block design. The significantly lowers scores for patients indicate a lower (visuo) spatial reasoning.

#### Executive functioning

*Inhibition* CG patients demonstrated significantly lower scores on the Stroop Inhibition, indicating a poor (response) inhibition. The comparable scores on the Stroop Interference indicate that patients do not have an increased sensitivity to interference.

*Cognitive Flexibility* Patients demonstrated lower scores on both the TMT B/A and Letterfluency. This indicates respectively that patients have an increased sensitivity to interference when it comes to cognitive flexibility and less flexibility in generating words. The comparable scores on all subtests of the WCST indicate that on a group level patients seem to be able to switch properly between strategies with a comparable amount of errors and perseverative responses.

#### Mental speed

The scores on TMT part A, which assesses visual and processing speed were comparable. The lower scores of patients on Stroop I & II, TMT part B indicate that patients needed more time to complete the tasks. On Symbol search and Substitution, which require processing speed, focusing attention and visual perception, patients had significantly lower scores.

### Cognitive functioning: NEPSY results

Considering the results of the NEPSY are expressed as percentile rank scores, these results are reported separately and shown in Table 3. The NEPSY results of seven children are quite comparable to the results of the older patients reported above. On the NEPSY, approximately half of the patients demonstrated scores below the reference range on inhibition tasks and predominantly scores below the reference range on cognitive flexibility tasks. The mental speed was also impaired considering all patients needed more time to complete the tasks, which may indicate that inhibitory demands slow down cognitive processing speed. Although patients needed more time, all completed the NEPSY Naming and Switching tasks in the reference range. The scores on the subdomain attention indicate a poor selective and sustained attention.

**Table 3:** NEPSY-II Results

NEPSY-II	*N*	Low percentile rank scores^a^ CG patients (*n*)	Normal percentile rank scores^b^ CG patients (*n*)
Executive functioning			
*Inhibition*			
- Naming Total Errors - Inhibition Total Errors - Switching Total Errors	7 7 5	≤2 (1), 3–10 (1), 11–25 (1) ≤2 (1), 11–25 (2) 3–10 (1), 11–25 (2)	4 4 2
*Cognitive flexibility*			
- Response Set, Total Correct	5	≤2 (1), 3–10 (1), 11–25 (1)	2
*Attention*			
- Auditory Attention, Total Correct	7	≤2 (2), 3–10 (1), 11–25 (2)	2
Mental Speed			
- Naming Total Completion Time - Inhibition Total Completion Time - Switching Total Completion Time	7 7 5	- 3–10 (2), 11–25 (5) -	7 - 5

Data reported in percentile rank scores. *CG* classical galactosemia

^a^low percentile rank scores: ≤2: well below the reference level, 3-10: below the reference level, 11-25: borderline / just below the reference level,

^b^normal percentile rank scores: 26-75: the reference level, >75: above the reference level

#### Cognitive functioning and initiation of treatment

There was no significant difference in the cognitive functioning results between patients in the pre-NBS group and early treated patients. The exclusion of the homozygous p.Ser135Leu patients (*n* = 2) did not change these results.

#### Cognitive functioning in relation to general intelligence

The cognitive test results were compared between patients with a low FSIQ (50–69), an intermediate FSIQ (70–85) and a normal FSIQ (> 85) (Table 2). On a group level, no significant differences were found on cognitive functioning between the FSIQ groups except for Symbol search (domain mental speed) and Block design (domain visuospatial functioning).

To evaluate if patients performed as expected based on their intellectual abilities, cognitive functioning was also individually evaluated in the context of the FSIQ. The results of the cognitive tests of adult and pediatric patients are shown separately (Tables 4 and 5). Of the adult patients, 4/19 had one and 1/19 had two worse than expected test scores. All patients had at least one better than expected test score. Of the pediatric patients, 4/17 patients had a worse than expected test score on at least one and at most three tests. A majority of the patients (12/17) had at least one better than expected test result.

**Table 4:**
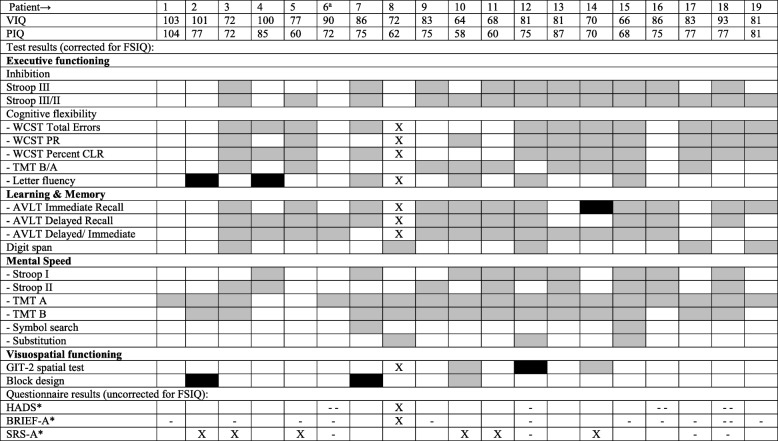
Individual Results, Adult Patients

^a^p.Ser135Leu homozygous patient. VIQ: Verbal IQ, PIQ: Performal IQ, FSIQ: Full Scale IQ, Stroop: Stroop Color Word Test, WCST: Wisconsin Card Sorting Test, PR: Perseverative Responses, CLR: Conceptual Level Responses, TMT: Trail Making Test, AVLT: Auditory Verbal Learning Test, GIT-II: Groninger Intelligentie Test 2, HADS: Hospital Anxiety and Depression Scale, BRIEF: Behavior Rating Inventory of Executive Function, SRS: Social Responsiveness Scale

X no test result, ■ test result worse than expected,  test result better than expected, □ test result as expected

*X no result, - - T-score on total scale in clinical range, - T-score on total scale in subclinical range, □ T-score on total scale within normal range.

**Table 5:**
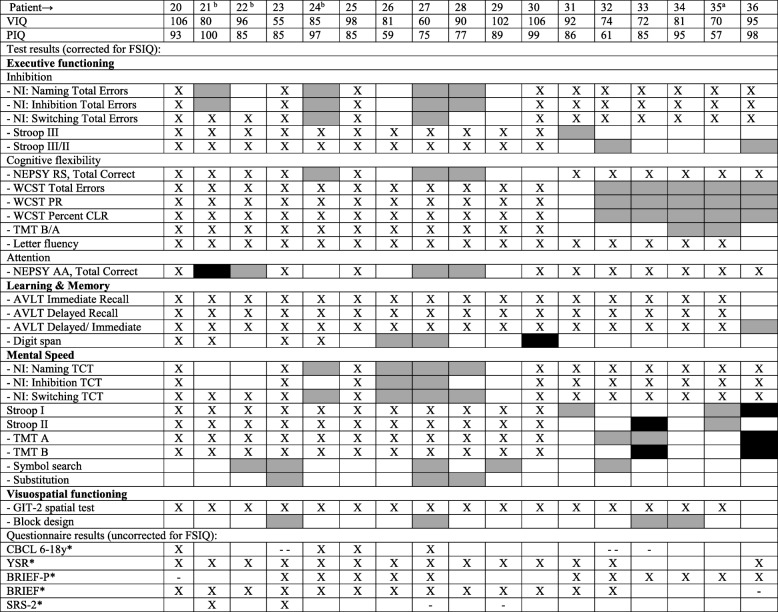
Individual Results, Pediatric Patients

^a^p.Ser135Leu homozygous patient, ^b^ Variant patient, VIQ: Verbal IQ, PIQ: Performal IQ, FSIQ: Full Scale IQ, NI: NEPSY Inhibition, Stroop: Stroop Color Word Test, RS: Response Set, AA: Auditory Attention, WCST: Wisconsin Card Sorting Test, PR: Perseverative responses, CLR: Conceptual Level Responses. TMT: Trail Making Test, AVLT: Auditory Verbal Learning Test, TCT: Total Completion Time, GIT-II: Groninger Intelligentie Test 2, CBCL 6–18y: Child Behavior Checklist 6–18 years, YSR: Youth Self Report, BRIEF: Behavior Rating Inventory of Executive Function, SRS: Social Responsiveness Scale

X no test result, ■ test result worse than expected, ▪ test result better than expected, □ test result as expected

*X no result, - - T-score on total scale in clinical range, - T-score on total scale in subclinical range, □ T-score on total scale within normal range

In total, 43% of the cognitive test results were better than expected when evaluated in the context of the FSIQ.

#### BRIEF (behavior rating inventory of executive function) questionnaire

Six parents completed the BRIEF-P questionnaire (data not shown). On the Behavioral Regulation Index (BRI), Metacognition Index (MI) and the Total scale, one parent reported T-scores in the subclinical range. Four adolescents completed the BRIEF questionnaire. On the BRI, MI and Total scale, one patient reported T-scores above 50, but well below the clinical range of 65.

Eighteen adults completed the BRIEF-A questionnaire. The median T-scores on the MI and Total Scale were higher in patients compared to normative data, but these differences were not significant after correction for multiple testing (*p* = .007). On the BRI, 7/18 patients (39%) scored in the subclinical range and 1/18 (6%) reached the clinical range. On the MI, 11/18 patients (61%) scored in the subclinical range and 3/18 (17%) in the clinical range. High scores on the MI were reported on all subdomains (initiative, memory, planning and organizing, task evaluation and tidiness). There was no significant correlation between the FSIQ and the BRI, MI and Total Scale on all used versions of the BRIEF (BRIEF-P, BRIEF and BRIEF-A).

### Behavioral functioning

#### CBCL (child behavior checklist) 6–18 years questionnaire

Parents reported scores in the subclinical and clinical range of the Internalizing Problems scale only (Table 6). On the subdomains of the Internalizing Problems scale, ‘withdrawn/ depressed’ and ‘somatic complaints’, parents reported significantly higher scores compared to normative data. No significant correlation between any of the CBCL outcome scales and FSIQ was found.

**Table 6:** CBCL 6–18y Results: Internalizing and Externalizing Problems and Social Functioning

CBCL 6–18y, syndrome scale	*N*	Results patients	Normative data	T ≥ 65 ≤ 68 (subclinical range)	T > 68 (clinical range)	*P*-value
CBCL	14					
- Internalizing problems		58.0 (33–72)	50			0.157
• Anxious/depressed • Withdrawn / depressed • Somatic complaints		54.0 (50–74) 58.0 (50–68) 54.5 (50–64)		*N* = 2 *N* = 3 *N* = 0	*N* = 1 *N* = 0 *N* = 0	**0.012** 0.005* 0.003*
- Externalizing problems		37.5 (33–62)				**0.018**
• Rule-Breaking behavior • Aggressive behavior		50.0 (50–60) 50.0 (50–62)		*N* = 0 *N* = 0	*N* = 0 *N* = 0	0.066 **0.042**
- WST		171 (150–201)	150	*N* = 2	*N* = 0	0.001*
• Withdrawn/ depressed • Social problems • Thought problems		58.0 (50–68) 56.5 (50–69) 51.0 (50–72)	50	*N* = 3 *N* = 1 *N* = 1	*N* = 0 *N* = 3 *N* = 1	0.005* 0.002* 0.007*

Data reported in T-scores, median (range). CBCL 6–18y: Child Behavior Checklist 6–18 years, WST: Withdrawn / depressed, Social problems & Thought problems. * Significant after Bonferroni-Holm correction

#### YSR (youth self report) questionnaire

A total of three adolescents completed the YSR questionnaire and did not report any problems on the Internalizing, Externalizing and Total problems scales.

#### HADS (hospital anxiety and depression) questionnaire

Eighteen adults, four males (22%) and 14 females (78%) with a median age of 25.5 years (18–47) completed the HADS (Table 7). The results of patients were compared to the norm data of 947 controls with a median age of 37.0 years (18–47) [17]. A higher percentage of patients reported scores indicative for an anxiety disorder and depression when compared to the reference group, but the difference was not statistically significant. All patients reporting a score of 8 or higher have a FSIQ between 70 and 85.

**Table 7:** HADS Results

HADS	*N*	Results patients	Total score ≥ 8 < 11 (%)	Total score ≥ 11 (%)	*N*	Results Reference group	Total score ≥ 8 < 11 (%)	Total score ≥ 11 (%)	*P*- value
	18				947				
- Anxiety scale - Depression scale		4.50 (1–16) 3.00 (0–13)	*N* = 1 (6%) *N* = 0 (0%)	*N* = 3 (17%) *N* = 1 (6%)		4.00 (0–17) 2.00 (1–19)	*N* = 109 (12%) *N* = 82 (9%)	*N* = 71 (7%) *N* = 38 (4%)	0.157 0.241

Data reported in median (range). HADS: Hospital Anxiety and Depression Scale

### Social functioning

#### SRS (social responsiveness scale)

In total, 38 patients completed the SRS (Table 8). Seven patients (18, 95%CI 11–36) had a T-score of ≥61 indicating a mild to moderate impaired social responsiveness. One patient (2.6, 95%CI 0.5–13.5) had a T-score of ≥76 indicating a severe disruption of social interaction in everyday life. Overall, the scores of patients were comparable to the normative data and individuals with elevated scores were within the expected frequency (based on a normal T-distribution the expected frequency of a score ≥ 61 is 16% and of a score ≥ 76 is 0.6%). The differences in T-scores between children and adults and males and females were not statistically significant. There was no significant correlation between the FSIQ and SRS-2 and FSIQ and SRS-A.

**Table 8:** SRS results

SRS	*N*	Results patients	Normative data	T ≥ 61 ≤ 75	T > 75	*P*-value
SRS-2 (parent)	23					
- Total		50.00 (40–85)	50	*N* = 3	*N* = 1	0.223
SRS-A (adult)	15					
- Total		53.00 (39–75)	50	*N* = 4	*N* = 0	0.116
SRS (all)	38					
- Total		52.50 (39–85)	50	*N* = 7	*N* = 1	0.073

Data reported in T-scores, median (range). SRS: Social Responsiveness Scale

#### CBCL 6–18 years, questionnaire

The T-scores on the subdomains 'withdrawn/ depressed', 's ocial problems' and 'thought problems', as well as its sum (WST) were significantly higher in patients when compared to normative data (Table 6). A minority of the parents reported scores in the subclinical and clinical range (on at least one and at most three domains).

### Possible confounders

To evaluate the effect of possible confounders, additional analyses were performed. Most pediatric patients underwent limited cognitive testing due to their age. Therefore, analyses were repeated without the data of patients who underwent the Wechsler Scale of Intelligence and just one additional cognitive test (i.e. the NEPSY), which did not change the previous reported results.

The exclusion of patients with comorbidity (dyslexia *n* = 2, ADHD *n* = 3, possible autism spectrum disorder *n* = 2, neonatal meningitis *n* = 2, skull fracture *n* = 1) and the exclusion of patients with scores indicative for an anxiety disorder or depression, did not change the previously reported results.

## Discussion

In this study, we aimed to investigate general intelligence, cognitive functioning on multiple domains, behavioral and social functioning in patients with CG. The results of this study demonstrate that as a group, CG patients have a below-average intelligence with a FSIQ of most patients between 70 and 85. Compared to normative data, patients demonstrated an overall impaired cognitive functioning, especially on tests within the domains mental speed, executive- and visuospatial functioning.

In our cohort, mental speed and visuospatial functioning were the most frequently affected cognitive domains. In previous studies, information processing speed (mental speed) has also been reported to be impaired [7, 8], but the finding that visuospatial functioning was affected contradicts with previous research [7–9]. Until now, the subdomains of the domain executive functioning were not investigated separately. In our cohort, overall scores indicated poor (response) inhibition and less (cognitive) flexibility in generating words, but patients were able to switch properly between tasks which requires cognitive flexibility as well. Considering patients in our cohort demonstrated less flexibility in generating words and both receptive and expressive language impairments have been reported in multiple case-studies, language should be further investigated. On the domain learning and memory, patients had more difficulty learning words, but once learned the immediate and delayed recall were relatively spared in our cohort. Previous studies indeed found no impairments on delayed and immediate recall [7–9], even though a subset of patients did perform on an impaired level [10]. In order to correctly interpreted the cognitive functioning results, it is important to evaluate them in the context of intellectual abilities. Considering the included patients demonstrated an overall below average intelligence and a correlation between IQ and cognitive performance has been demonstrated [18], the cognitive outcomes were compared between patients with a low FSIQ (50–69), an intermediate FSIQ (70–85) and a normal FSIQ (> 85). Interestingly, no significant differences in cognitive functioning between the three groups were found with the exception of two tests (block design and symbol search) that directly contribute to the FSIQ itself.

Considering the large differences in cognitive functioning between patients within the same FSIQ group, individual test results on cognitive functioning were re-evaluated in the context of intellectual abilities, which revealed that patients’ cognitive outcomes may exceed IQ based expectations. This emphasizes the general idea that the FSIQ is an ultimate resultant that may not reflect underlying specific qualities and vulnerabilities of the individual patient, and that a more comprehensive neuropsychological evaluation will provide a better insight in one’s strengths and weaknesses. According to the literature, especially executive functioning is crucial for academic performance and has a predictive value for academic achievement [19], which could also explain the higher educational attainment in our patients than their FSIQ would suggest.

Even though we investigated cognitive functioning on multiple domains with multiple tests and evaluated scores in context of the intellectual abilities, the large variability in cognitive functioning remained and a clear profile could not be distinguished. This variability complicates the interpretation of results and makes it impossible to draw an overall conclusion on cognitive functioning in CG. This finding underlines the need for an individual assessment in all patients. The large intra-individual variability in cognitive functioning has been demonstrated in healthy adults as well and the question remains whether found abnormalities indeed indicate the presence of brain dysfunction [20]. Therefore results should be evaluated in the context of daily functioning of the individual patient.

The VIQ and PIQ were evaluated as well. Overall, patients demonstrated a slightly higher VIQ than PIQ, which is in line with previous studies [8, 21]. Considering the broad standard deviations and confidence intervals and small differences between VIQ and PIQ on a group level, it cannot be concluded that verbal skills (as numerically measured by VIQ) are better than non-verbal skills (PIQ). In those patients with a significantly higher VIQ however, this could potentially lead to an overestimation of the patients’ abilities due to relatively good verbal skills. This is a relevant finding because in daily life this may put patients at risk for excessive demands in relation to their more limited cognitive abilities.

On the BRIEF questionnaires, only a minority of the already few parents reported executive functioning problems in the subclinical and clinical range which is in contrast to a vast majority of the adult patients. This may be explained by the fact that the children of these parents perform relatively well.

On the CBCL, all reported scores in the subclinical and clinical range were reported on the internalizing problems scale. Interestingly, on the YSR self-report, adolescents reported no problems even though the parents of two out of three patients reported scores in the clinical range on the internalizing problems scale. This is in line with a previous study which demonstrated that parents reported more problems than children, who might not experience or recognize problems in their functioning [9]. On the subscales of the CBCL indicative for social functioning (WST), CG parents reported statistically significantly higher problem scores than population norms. However, only a minority of parents reported scores in the subclinical or clinical range. Scores on the SRS were comparable to normative data and elevated scores indicating problems in social functioning were only reported by a minority of the patients. Importantly, our study did not find increased levels of social irresponsiveness or features suggestive of autism in CG. This is an important finding with regard to the observation that CG patients would manifest autistic traits. Future research is needed to understand poor social functioning in CG other than from deficits in the autism spectrum. A recent study found impaired visual information processing and facial emotion recognition in CG patients, which might contribute to the difficulties in social interactions observed in patients [22].

On the HADS, CG patients reported higher scores on the anxiety and depression scales than the reference group. The fact that this difference was not statistically significant might be due to the small numbers of patients included in this study. Since CG patients may be at risk for anxiety and depression, routine screening is warranted.

All but one patient reporting problems on the BRIEF and all patients with elevated scores on the HADS had a FSIQ between 70 and 85, suggesting that these patients may be at risk to develop problems in everyday life. The fact that these problems were not reported by patients with a FSIQ below 70 could indicate that they do not experience problems, are not aware of problems or simply have more difficulty in expressing problems. Another explanation could be that these patients are protected and guided more in everyday life or that the questionnaires used are not suited for patients with lower intellectual abilities.

Besides patients with classic phenotypes, our cohort also included four NBS detected variant patients and two patients with a homozygous p.Ser135Leu mutation. The four variant patients received limited testing due to their age. Even though these patients are still young, they demonstrated a FSIQ above 85 and scores on the cognitive tests in the normal range. Follow-up of these patients is needed before it can be concluded that these patients indeed have a better neurocognitive outcome than patients with classical phenotypes. Patients with a homozygous p.Ser135Leu genotype are expected to have residual GALT enzyme activity in different tissues, which may improve their outcome. However, our two patients had a FSIQ of 71 and 61 and below average to low scores on several cognitive tests. These two patients did not present with critical illness in the neonatal period and were diagnosed late which resulted in a prolonged exposure to galactose which might explain this finding.

In our cohort, an early initiation of the diet because of NBS or family screening did not result in a higher FSIQ nor higher scores on the cognitive tests. Since most of the early treated patients are young and therefore received limited neuropsychological testing, follow-up is warranted before definitive conclusions can be drawn.

At this time it is unclear whether neurocognitive functioning declines with age as was suggested by cross-sectional studies [8, 23, 24], but contradicted by longitudinal studies [5, 21]. In our cross-sectional study, age was negatively correlated with FSIQ. The exclusion of the younger variant patients in our cohort with an expected better clinical outcome, resulted in a non-significant correlation. Moreover, neurocognitive decline should be assessed in longitudinal studies.

### Limitations

Not all CG patients visiting our expertise outpatient clinic underwent a neuropsychological assessment. Even though the patient group who chose not to undergo a neuropsychological assessment contains both patients with normal cognitive functioning and patients with an impaired cognitive functioning reported in their medical charts, this may cause selection bias.

The data presented in this paper should be interpreted with care because a small number of patients provides statistical challenges. In adults, most T-scores on cognitive functioning tests are corrected for educational attainment, which might favor the results of the patients since they perform on a lower education level compared to the general population. However, significantly lower scores were still shown in patients when compared to the general population. Since patients have a substantially lower FSIQ compared to the general population, the individual results on cognitive functioning tests were evaluated in the context of the FSIQ of patients. This is indeed somewhat superficial, as the FSIQ arises from subtests of cognitive abilities that correlate with one another, and with the neuropsychological tests. Therefore, this controlling for consistent variables gives rise to positive manifold and may overshadow relevant cognitive impairments. Also, an interesting observation is that general intelligence appears to account for a larger share of cognitive variance in individuals exhibiting lower intelligence (as measured by IQ or mental age) than in individuals exhibiting higher intelligence [25].

In this study we did not intend to investigate the constructs of cognitive functioning. In order to assess cognitive functioning on multiple domains, the categorization of the domains as proposed by other studies was used [10]. The division in domains is needed to properly investigate cognitive functioning, but it is important to be aware that cognitive functioning tests may overlap between domains.

Longitudinal studies are needed to investigate apparent age-related dynamic changes between the different scales of intelligence as measured by the Wechsler scales. Moreover, re-assessment with latest iterations of the Wechsler scales will provide practitioners and scientist with more conceptual and practical insight into the developmental processes and the complex concept of intelligence in CG.

Besides intelligence, cognitive functioning, behavior and social functioning, there are other factors such as adaptive skills, personal, family and environmental factors that influence functioning of individuals to a certain extent and lay outside the scope of this article.

Adult patients completed the questionnaires during the neuropsychological assessment, whereas most parents completed the questionnaires at home. This resulted in a limited number of returned questionnaires completed by parents. The self-reported questionnaires might be hard on patients with an intellecutal deficiency. Especially the SRS was difficult for patients with a FSIQ below 70 and therefore the results of these patients were not reported.

### Strengths

In this study, we included all patients irrespective of their expected neuropsychological outcome and excluded patients with a second (genetic) diagnosis, which could influence neuropsychological functioning. Therefore, this is not only a relatively large but also a representative CG cohort.

The assessment of cognitive functioning on specific domains with the use of multiple tests per domain provides a more reliable insight into the neuropsychological functioning of CG patients, than when only one test per domain is administered. The results of pediatric and adult patients were combined where possible. Since most pediatric patients received limited testing due to their age, analyses were repeated after the exclusion of these patients. The exclusion of these patients did not change the results and did not provide a more distinctive neuropsychological profile.

Since comorbidity, such as ADHD, autism, neonatal meningitis and dyslexia may cause executive functioning impairment [26] and anxiety and depression might be related to cognitive impairment and executive dysfunctioning in particular [27], analyses were repeated after the exclusion of these patients, however this did not change the results.

## Conclusions

The current study provides insights in general intelligence, functioning on multiple cognitive domains, behavior and social functioning of patients with CG. As a group, patients have a substantially lower IQ and impaired cognitive functioning when compared to the general population and may be at risk for internalizing problems. Importantly, individual differences are considerable and specific cognitive abilities may exceed expectations that are based on the IQ. Based on the findings of our study, an individual neuropsychological assessment including the evaluation of behavior and social functioning is advised in all CG patients. In order to provide patients with timely and optimal support, the results of the neuropsychological assessment should be evaluated in a broader context, which includes adaptive functioning, the support system, the educational level and the capacity of patients and should include follow-up. This to ensure patients can reach their full potential without being subjected to excessive cognitive and emotional demands.

## Methods

### Patients and recruitment

All pediatric and adult patients with CG, visiting our multidisciplinary galactosemia expertise outpatient clinic, were offered a standardized neuropsychological assessment as part of patient care according to the International guideline for CG patients [28]. Adult patients (≥18 years) or parents of patients (< 18 years) were asked to complete the Social Responsiveness Scale (SRS). CG patients who are treated in other metabolic centers but participated in research in our outpatient clinic and recently received a neuropsychological assessment, data were retrieved after informed consent and incorporated if admissible.

#### Inclusion and exclusion criteria

All patients with erythrocyte GALT activity < 15% of the reference mean and/or two known pathogenic variations in the *GALT* gene were eligible for participation in this study.

All patients with a second genetic diagnosis influencing clinical outcome were excluded. The results on cognitive functioning of patients with a FSIQ below 50 were excluded. The SRS of adult patients with an unknown FSIQ or a FSIQ below 70 were excluded.

### Neuropsychological measures

The comprehensive neuropsychological assessment is summarized in Additional file 1: Table S8 and includes standardized tests that cover:

- General intelligence
- Cognitive functioning on the domains: learning and memory, visuospatial functioning, executive functioning and mental speed.
- Questionnaires (self- and proxy reported) on executive functioning, behavior and social functioning

In children, Wechsler’s Verbal IQ (VIQ) and in adults Wechsler’s Verbal Comprehension Index (hereafter VIQ) indicates verbal functioning. In children, Wechsler’s Performance IQ (PIQ) and in adults Wechsler’s Perceptual Reasoning Index (hereafter PIQ) indicates non-verbal functioning.

Since it has been demonstrated that the subscales Withdrawn / depressed, Social problems and Thought problems (WST) of the Child Behavior Checklist (CBCL) 6–18y can be used to assess social functioning [29, 30], these subscales and its sum were evaluated as well.

### Data collection

The results of the neuropsychological assessment and data on educational attainment were collected and stored in an electronic clinical report form in Castor Electronic Data Capture, a good clinical practice compliant data management system [31].

### Statistical analysis

SPSS version 25.0 (SPSS Inc. Chicago, Illinois, USA) was used to perform all statistical analyses. Descriptive analyses included means and standard deviations if data was normally distributed and median and ranges in case of a non-normal distribution. General intelligence was standardized to an IQ-score (mean 100, *SD*15). All scores on cognitive functioning tests and questionnaires were standardized to T-scores (mean 50, *SD*10), except for the HADS (Hospital Anxiety and Depression Scale) which is scored according to a Likert scale (0–3) resulting in a total score and the Developmental NEuroPSYchological Assessment (NEPSY) scores, which are expressed as percentile rank (pr) scores, ranging from well below the reference level (pr < 2) to above the reference level (pr > 75). Depending on the cognitive tests used, the standardized T-scores were corrected for age and/or gender and in adults most cognitive tests were corrected for educational attainment as well. The T-scores of patients were compared to normative data as reflection of the general population (T-score 50) with the use of the non-parametric sign test. A preliminary analysis showed a large variability in the FSIQ of patients. The effect of the FSIQ on cognitive functioning was evaluated by dividing patients into three FSIQ groups; group 1: FSIQ 50–69, group 2: FSIQ 70–85 and group 3: FSIQ > 85. Hereafter, individual cognitive test results were re-evaluated in the context of the FSIQ. More specifically, the FSIQ was converted into a T-score and was compared to the T-scores on the cognitive tests. In case a patient scored -1*SD* (T-score ≤ 10) beneath their expected T-score, the cognitive test score was considered ‘worse than expected’ and a score above +1*SD* (T-score ≥ 10) was defined as ‘better than expected’ based on the FSIQ. Differences between groups were tested with the Kruskal Wallis test or Mann-Whitney U- test where appropriate. For the Hospital Anxiety and Depression Scale (HADS), the results of the patients were compared to the norm data of a reference group [17]. The Spearman’s rank coefficient test was used to test for correlations and in case of a significant correlation, linear regression was performed. To evaluate the effect of possible confounders on our data, additional analyses were performed after the exclusion of patients with very limited data and patients with comorbidity potentially affecting cognitive functioning. To evaluate the effect of an early initiation of the diet on neurocognitive outcome, patients diagnosed before the introduction of newborn screening (pre-NBS) with a clinical presentation were compared to patients with an early diagnosis based on NBS or family screening (because of an older sibling with CG). *P*-values below 0.05 were considered statistically significant. Multiple tests regarding a single hypothesis were corrected with the Bonferroni-Holm correction.

## Supplementary information

**Additional file 1 : Table S8**. The Neuropsychological Assessment

## Data Availability

The data that support the findings of this study are available from the corresponding author upon reasonable request.

## Abbreviations

AVLT: Auditory verbal learning test
BRI: Behavioral regulation index
BRIEF: Behavior rating inventory of executive function
CBCL 6–18y: Child behavior checklist 6–18 years
CG: Classical galactosemia
FSIQ: Full scale intelligence quotient
GALT: Galactose-1-phosphate uridyltransferase
GIT_II: Groninger Intelligentie Test 2
HADS: Hospital Anxiety and Depression Scale
MI: Metacognition Index
NBS: Newborn screening
NEPSY: Developmental NEuroPSYchological Assessment
PIQ: Performance IQ
PR: Percentile rank
SRS: Social responsiveness scale
STROOP: Stroop color word test
TMT: Trail Making Test
VIQ: Verbal IQ
WAIS: Wechsler adult intelligence scale
WCST: Wisconsin card sorting test
WISC: Wechsler intelligence scale for children
WPPSI: Wechsler Preschool and Primary Scale of Intelligence
WST: Withdrawn / depressed, Social problems & Thought problems
YSR: Youth Self Report
