# Correction to: Classical galactosemia: neuropsychological and psychosocial functioning beyond intellectual abilities

**DOI:** 10.1186/s13023-020-01447-z

**Published:** 2020-09-07

**Authors:** Mendy M. Welsink-Karssies, Kim J. Oostrom, Merel E. Hermans, Carla E. M. Hollak, Mirian C. H. Janssen, Janneke G. Langendonk, Esmee Oussoren, M. Estela Rubio Gozalbo, Maaike de Vries, Gert J. Geurtsen, Annet M. Bosch

**Affiliations:** 1 Department of Pediatrics, room H7-270, Amsterdam University Medical Centre, MC, PO BOX 22660, 1100 DD Amsterdam, The Netherlands; 2 Psychosocial Department, Emma Children’s Hospital, Amsterdam UMC, University of Amsterdam, Amsterdam, The Netherlands; 3 Department of Medical Psychology, Amsterdam UMC, University of Amsterdam, Amsterdam, The Netherlands; 4 Department of Internal Medicine, Division of Endocrinology and Metabolism, Amsterdam UMC, University of Amsterdam, Amsterdam, The Netherlands; 5 Department of Internal Medicine, Radboud University Medical Center, Nijmegen, The Netherlands; 6 Department of Internal Medicine, Center for Lysosomal and Metabolic Diseases, Erasmus MC, University Medical Center Rotterdam, Rotterdam, The Netherlands; 7 Department of Pediatrics, Center for Lysosomal and Metabolic Diseases, Erasmus MC, University Medical Center Rotterdam, Rotterdam, The Netherlands; 8 Department of Pediatrics and Department of Clinical Genetics, Maastricht University Medical Center, Maastricht, The Netherlands; 9 Department of Pediatrics, Radboud University Medical Center, Nijmegen, The Netherlands

**Correction to: Orphanet Journal of Rare Diseases (2020) 15:42**

**https://doi.org/10.1186/s13023-019-1277-0**

Following the original article’s publication [1] the authors informed us of the following errors:

1. Author M. Estela Rubio Gozalbo’s first and last names were captured incorrectly. The author’s first names are M. Estela, while last names are Rubio Gozalbo.

The correct author’s name has been updated in the original article [1] and shown in the author list of this Correction.

2. In Table 2 ‘Digit span’ and ‘GIT-2’ are tests and should be preceded by a - just like the other tests in the table.

Furthermore, ‘Responses’ is not a domain and should therefore be deleted.

Finally, ‘Cconceptual’ should be corrected to ‘conceptual’.

The table is shown here corrected.

**Table 2:** Cognitive Functioning Results

Domain	*N*	Results patients	*P*-value^a^	*N*	FSIQ 50–69 (Group 1)	*N*	FSIQ 70–85 (Group 2)	*N*	FSIQ > 85 (Group 3)	*P*-value^b^
Learning & Memory										
- AVLT Immediate Recall	19	46.0 (9–61)	**0.029**	4	40.0 (9–46)	11	45.00 (35–61)	4	49.50 (47–51)	0.121
- AVLT Delayed Recall	19	46.0 (15–65)	0.545	4	49.5 (15–58)	11	46.00 (34–65)	4	50.00 (43–55)	0.956
- AVLT Delayed / Immediate	19	52.0 (38–65)	0.445	4	55.5 (43–62)	11	52.00 (38–65)	4	52.00 (39–62)	0.803
- Digit span	35	43.0 (20–63)	**< 0.0005**^**c**^	8	30.00 (20–57)	15	43.00 (27–60)	12	48.50 (33–63)	**0.017**
Visuospatial functioning										
- GIT-2^a^ spatial test	19	36.0 (23–50)	**< 0.0005**^**c**^	4	26.5 (23–40)	11	35.0 (28–40)	4	41.5 (40–50)	**0.019**
- Block design	42	38.5 (20–53)	**< 0.0005**^**c**^	9	30.0 (20–40)	17	37.0 (27–50)	16	37.0 (33–53)	**< 0.0005**^**c**^
Executive functioning										
*Inhibition*										
- Stroop III (Inhibition)	25	45.0 (20–56)	**0.003**^**c**^	6	27.0 (20–49)	13	48.00 (22–56)	6	46.0 (35–53)	0.078
- Stroop III/II (Interference)	25	49.0 (30–66)	0.537	6	43.0 (30–60)	13	50.00 (31–66)	6	47.5 (40–63)	0.642
*Cognitive flexibility*										
- WCST Total number of errors	24	50.5 (27–67)	0.988	6	46.0 (27–50)	12	51.50 (37–67)	6	52.0 (39–64)	0.134
- WCST Perseverative responses	24	51.0 (30–81)	0.626	6	46.0 (33–52)	12	53.00 (30–81)	6	52.0 (35–73)	0.278
- WCST Percent conceptual level responses	24	49.5 (27–64)	0.951	6	48.0 (27–51)	12	52.00 (39–64)	6	51.0 (37–62)	0.270
- TMT B/A	25	44.0 (27–57)	**0.002**^**c**^	6	45.0 (27–50)	13	43.00 (27–57)	6	48.5 (40–57)	0.510
- Letter fluency	19	37.0 (27–67)	**0.001**^**c**^	4	31.0 (28–38)	11	39.00 (31–67)	4	34.0 (27–56)	0.143
Mental Speed										
- Stroop I (Color naming)	25	40.0 (25–61)	**0.001**^**c**^	6	35.0 (25–43)	13	43.00 (35–61)	6	46.5 (33–55)	0.077
- Stroop II (Word reading)	25	37.0 (20–56)	**< 0.0005**^**c**^	6	30.0 (20–40)	13	43.00 (20–56)	6	39.0 (33–56)	0.063
- TMT A (Digit sequencing)	25	52.0 (20–67)	**0.352**	6	50.5 (20–59)	13	56.00 (43–67)	6	47.5 (33–67)	0.173
- TMT B (Digit-Letter-Switching)	25	45.0 (20–58)	**0.003**^**c**^	6	33.5 (20–47)	13	46.00 (20–58)	6	45.0 (33–56)	0.111
- Symbol search	41	43.0 (20–67)	**< 0.0005**^**c**^	9	23.0 (20–50)	17	40.00 (27–67)	15	47.0 (40–60)	**0.001**^**c**^
- Substitution	42	40.0 (23–57)	**< 0.0005**^**c**^	9	30.0 (23–47)	17	40.00 (30–57)	16	43.0 (33–53)	**0.006**

Data reported in T-scores, median (ranges). ^a^ Patient data vs. normative data (T-score 50), ^b^ Comparison between FSIQ groups, ^**c**^ Significant after Bonferroni-Holm correction. *FSIQ* full scale IQ, *AVLT* auditory verbal learning test, *GIT-II* groninger intelligentie test 2, *Stroop* stroop color word test, *WCST* wisconsin card sorting test, *TMT* trail making test

3. The grey box for ‘test result better than expected’ is missing from the legends of Tables 4 and 5.

The two tables with their respective legends are shown here corrected.

**Table 4:**
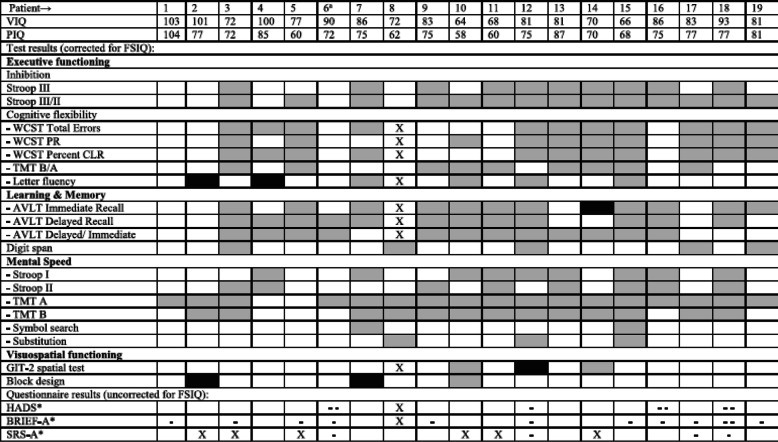
Individual Results, Adult Patients

^a^p.Ser135Leu homozygous patient. *VIQ* Verbal IQ, *PIQ* Performal IQ, *FSIQ* Full Scale IQ, *Stroop* Stroop Color Word Test, *WCST* Wisconsin Card Sorting Test, *PR* Perseverative Responses, *CLR* Conceptual Level Responses, *TMT* Trail Making Test, *AVLT* Auditory Verbal Learning Test, *GIT-II* Groninger Intelligentie Test 2, *HADS* Hospital Anxiety and Depression Scale, *BRIEF* Behavior Rating Inventory of Executive Function, *SRS* Social Responsiveness Scale

X no test result, ■ test result worse than expected,  test result better than expected, □ test result as expected

*X no result, - - T-score on total scale in clinical range, - T-score on total scale in subclinical range, □ T-score on total scale within normal range

**Table 5:**
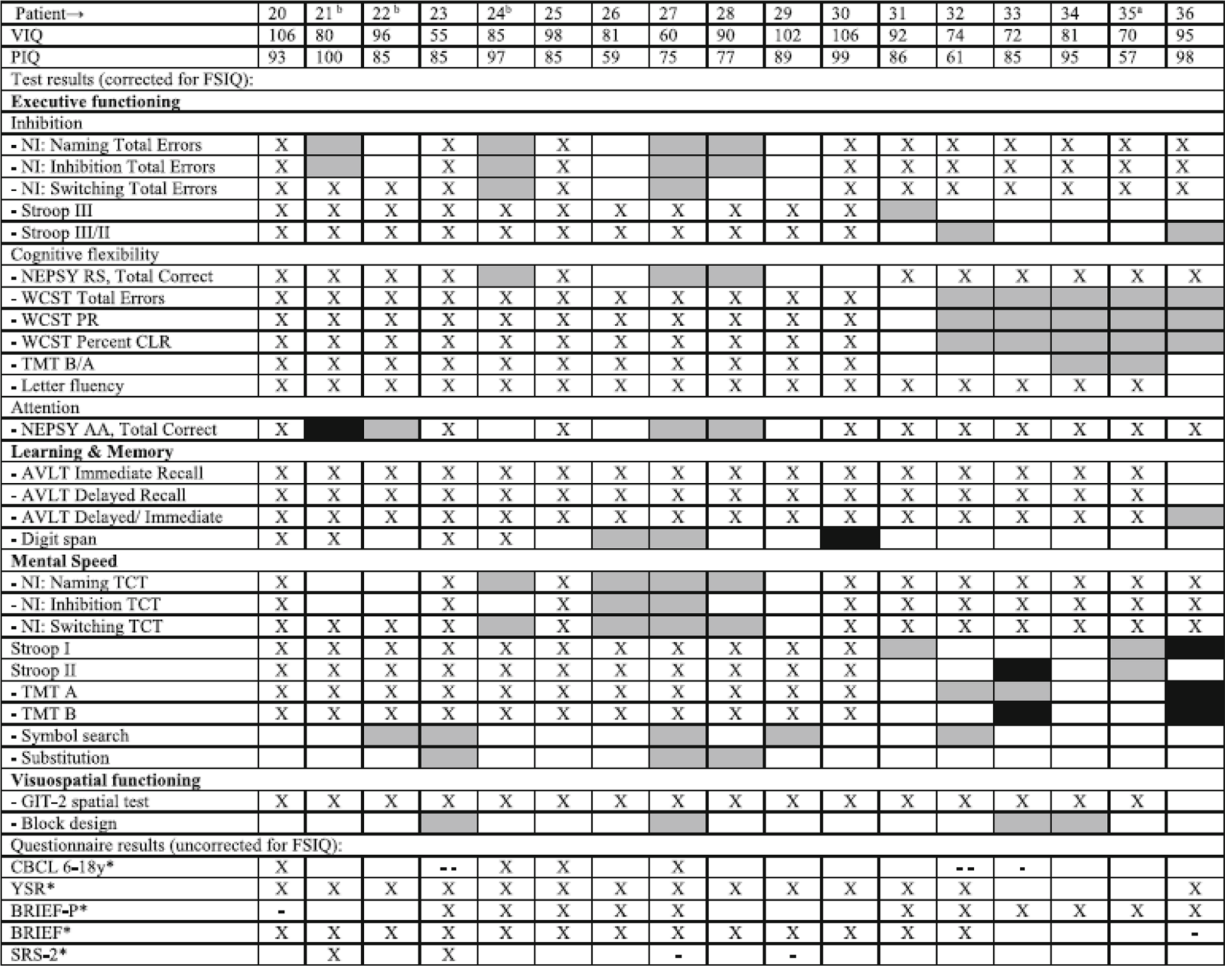
Individual Results, Pediatric Patients

^a^p.Ser135Leu homozygous patient, ^b^ Variant patient, *VIQ* Verbal IQ, *PIQ* Performal IQ, *FSIQ* Full Scale IQ, *NI* NEPSY Inhibition, *Stroop* Stroop Color Word Test, *RS* Response Set, *AA* Auditory Attention, *WCST* Wisconsin Card Sorting Test, *PR* Perseverative responses, *CLR* Conceptual Level Responses, *TMT* Trail Making Test, *AVLT* Auditory Verbal Learning Test, *TCT* Total Completion Time, *GIT-II* Groninger Intelligentie Test 2, *CBCL 6–18y* Child Behavior Checklist 6–18 years, *YSR* Youth Self Report, *BRIEF* Behavior Rating Inventory of Executive Function, *SRS* Social Responsiveness Scale

X no test result, ■ test result worse than expected,  test result better than expected, □ test result as expected

*X no result, - - T-score on total scale in clinical range, - T-score on total scale in subclinical range, □ T-score on total scale within normal range

4. In Table S8 of the Additional file 1, some rows have shifted. ‘Anxiety & Depression’ and ‘Social Functioning’ should be shown on separate lines. HADS should be in line with ‘Anxiety & Depression’ and SRS with ‘Social Functioning’.

In addition, subsequent references should be renumbered as 35–49.

The corrected Additional file 1 accompanies this Correction.

## Supplementary information

**Additional file 1: Table S8.** The Neuropsychological Assessment.
