# Supplementary material for: Classical galactosemia: neuropsychological and psychosocial functioning beyond intellectual abilities
Source: Orphanet J Rare Dis. 2020 Feb 7;15:42. doi: 10.1186/s13023-019-1277-0 (PMC7007688; doi:10.1186/s13023-019-1277-0)
Supplement: Supplementary file 1 — Additional file 1 : Table S8. The Neuropsychological Assessment [file 13023_2019_1277_MOESM1_ESM.docx]

**Supplementary material**

Table 8. The Neuropsychological Assessment

| Age | 2y;6m - 4y;11m | 5y - 11y;11m | 12y - 16y;11m | 17y | ≥ 18y |
| --- | --- | --- | --- | --- | --- |
| General intelligence (n) | - WPPSI-III^NL^ (2) | - WPPSI-III^NL^ (5)  - WISC-III^NL^ (12) | WISC-III^NL^ (7) | WAIS-IV^NL^ (1) | WAIS-IV^NL^ (21) |
| Cognitive functioning (*tests*) | Block design | NEPSY-II  Symbol search  Substitution  Block design | Stroop  WCST  TMT  Digit span  Symbol comparing  Substitution  Block design | Stroop  WCST  TMT  Letter Fluency  AVLT  Digit span  Symbol search  Symbol substitution coding  GIT-II spatial test  Block design | Stroop  WCST  TMT  Letter Fluency  AVLT  Digit span  Symbol search  Symbol substitution coding  GIT-II spatial test  Block design |
| *Questionnaires* | | | | | |
| Executive functioning  Behavior   - Anxiety & depression Social Functioning | CBCL 1,5-5 (p)  SRS-2 (p) (≥ 4y) | BRIEF-P (p)  CBCL 1,5-5 (p)/  CBCL 6-18 (p)  SRS-2 (p) | BRIEF (c)  CBCL 6-18 (p)  YSR (c)  SRS-2 (p) | BRIEF (c)  CBCL 6-18 (p)  YSR (c)  SRS-2 (p) | BRIEF-A (a)  HADS (a)  SRS-A (a) |

*Notes.* NL: Dutch version, y = years, m = months, p = reported by parents, c = self-reported by child, a = self-reported by adult

*-* General intelligence; the Wechsler Scales of Intelligence (WSI): the Wechsler Preschool and Primary Scale of Intelligence (WPPSI), completed by children ≥ 2y;6m until the 1st & 2nd grade of primary education (32), the Wechsler Intelligence Scale for Children (WISC), completed by children ≥ 3rd grade of primary education (33), the Wechsler Adult Intelligence Scale (WAIS) (34, 35). Variables of interest: Full Scale IQ (FSIQ), Verbal IQ (VIQ), Performal IQ (PIQ). Subtests used for cognitive functioning: Symbol search (symbol comparing), Substitution (symbol substitution coding), Digit span, Block design.

*-* Cognitive functioning (tests); Developmental NEuroPSYchological Assessment (NEPSY) (36), Stroop Color Word Test (Stroop) (37), Wisconsin Card Sorting Test (WCST) (38), Trail Making Test (TMT) (39), Letter Fluency (40), Auditory Verbal Learning Test (AVLT) (41), GIT-II spatial test (42).

*-* Questionnaires (self- and proxy reported):

- Executive functioning: BRIEF (Behavior Rating Inventory of Executive Function): BRIEF-P (43), BRIEF (44), BRIEF-A (45).

- Behavior: CBCL (Child Behavior Checklist) 6-18y (46), YSR (47) and HADS (Hospital Anxiety and Depression Scale) (48)

- Social Functioning: SRS (Social Responsiveness Scale): SRS-2 (49), SRS-A (50).
